# Prognostic Value of EZH2 in Non-Small-Cell Lung Cancers: A Meta-Analysis and Bioinformatics Analysis

**DOI:** 10.1155/2020/2380124

**Published:** 2020-11-09

**Authors:** Kui Fan, Chuan-long Zhang, Yuan-fu Qi, Xin Dai, Yoann Birling, Zhao-feng Tan, Fang Cao

**Affiliations:** ^1^College of Traditional Chinese Medicine, Shandong University of Traditional Chinese Medicine, Jinan, Shandong, China; ^2^Department of Oncology, Affiliated Hospital of Shandong University of Traditional Chinese Medicine, Jinan, Shandong, China; ^3^NICM Health Research Institute, Western Sydney University, Westmead, Australia

## Abstract

**Background:**

The prognosis of non-small-cell lung cancer (NSCLC) has not been significantly improved. In the past several years, research on epigenetics is in full swing. There is a focus on the gene EZH2; however, its role as a predictor of the prognosis of NSCLC is in the debate.

**Objective:**

To clarify if the expression level of EZH2 can influence the prognosis of NSCLC and explain its prognostic value.

**Methods:**

We have systematically searched PubMed, Web of Science, and Cochrane library, screened relevant articles, and conducted a meta-analysis on the expression level of EZH2 in NSCLC. We collected the hazard ratio (HR) and the 95% confidence interval (CI) and used STATA 12.0 to calculate the combined result of EZH2 overall survival. In addition, we conducted subgroup analyses, a sensitivity analysis, and a funnel plot to test the reliability of the results. We further validated these meta-analysis results using the Kaplan-Meier plotter database and The Cancer Genome Atlas (TCGA) database. In addition, we have investigated the correlation between EZH2 expression and EGFR expression, KRAS expression, BRAF expression, and smoking in TCGA database to further explore the mechanism behind the influence of high EZH2 expression on lung cancer prognosis.

**Results:**

13 studies including 2180 participants were included in the meta-analysis. We found that high expression of EZH2 indicates a poor prognosis of NSCLC (HR = 1.65 and 95% CI 1.16-2.35; *p* ≤ 0.001). Subgroup analyses showed high heterogeneity in stages I-IV (*I*^2^ = 85.1% and *p* ≤ 0.001) and stages I-III (*I*^2^ = 66.9% and *p* = 0.029) but not in stage I (*I*^2^ = 0.00% and *p* = 0.589). In the Kaplan-Meier plotter database, there was a high expression in 963 cases and low expression in 964 cases (HR = 1.31 and 95% CI 1.15-1.48; *p* < 0.05). Further analysis found that the high expression of EZH2 was statistically significant in lung adenocarcinoma (HR = 1.27and 95% CI 1.01−1.6; *p* = 0.045), but not in lung squamous cell carcinoma (HR = 1.03 and 95% CI 0.81−1.3; *p* = 0.820). The results of the TCGA database showed that the expression of EZH2 in normal tissues was lower than that in lung cancer tissues (*p* < 0.05). Smoking was associated with high expression of EZH2 (*p* < 0.001). EZH2 was also highly expressed in lung cancers with positive KRAS expression, and the correlation was positive in lung adenocarcinoma (*r* = 0.3129 and *p* < 0.001). The correlation was also positive in lung squamous cell carcinoma (*r* = 0.3567 and *p* < 0.001). EZH2 expression was positively correlated with BRAF expression (*r* = 0.2397 and *p* < 0.001), especially in lung squamous cell carcinoma (*r* = 0.3662 and *p* < 0.001). In lung squamous cell carcinoma, a positive yet weak correlation was observed between EZH2 expression and EGFR expression (*r* = 0.1122 and *p* < 0.001).

**Conclusions:**

The high expression of EZH2 indicates a poor prognosis of NSCLC, which may be related to tumor stage or cancer type. EZH2 may be an independent prognostic factor for NSCLC. EZH2 high expression or its synergistic action with KRAS and BRAF mutations affects the prognosis of non-small-cell lung cancer.

## 1. Introduction

Lung cancer is the cancer that has the highest incidence and mortality rate worldwide [1, 2]. NSCLC accounts for 80% to 85% of all lung cancer cases [3]. In recent years, great progress in the diagnosis and treatment of NSCLC has been achieved, yet the overall five-year survival rate is still less than 21% [4]. A lot of evidence shows that epigenetic regulation plays an important role in the occurrence and development of tumors. Epigenetic modification can regulate chromatin status and gene expression through pathways such as DNA methylation and demethylation, histone modification, and chromatin remodeling without changing the DNA sequence [5, 6]. Enhancer of zeste homolog 2 (EZH2) is an evolutionarily conserved gene that participates in a variety of biological functions (e.g., cell cycle, cell proliferation, and cell differentiation). EZH2 is a key factor of tumor growth and metastasis [7–9].

Recent studies suggest that EZH2 has a potential prognostic role in patients with NSCLC. However, based on their results, the prognostic value of EZH2 expression levels in NSCLC is controversial. It is generally believed that meta-analysis is a powerful statistical tool that can overcome the limitations of different individual research sample sizes and produce the best estimates. Therefore, we collected all eligible published studies and performed a meta-analysis to quantify the prognostic value of pretreatment EZH2 in NSCLC.

## 2. Materials and Methods

### 2.1. Search Strategy

We conducted a meta-analysis according to the PRISMA guidelines [10]. We searched the online databases PubMed, Web of Science, and Cochrane Library for every eligible study until February 2020.

**Figure 1:**
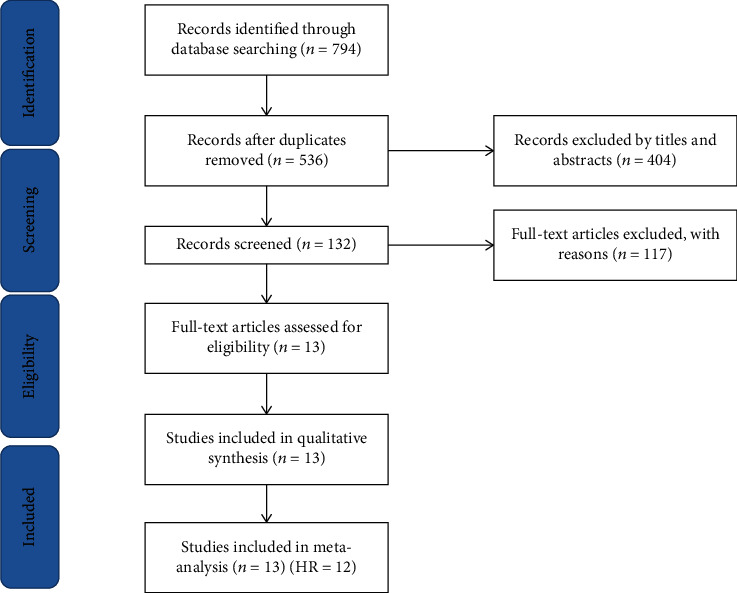
Flow diagram of the study selection process. HR: hazard ratio.

The search terms were “Enhancer of zeste homolog 2” OR “EZH2” OR “ENX-1” AND “lung cancer” OR “lung adenocarcinoma” OR “lung tumors” OR “NSCLC” OR “LAD” OR “ADC.” We identified duplicates using the authors' name, institution, clinical trial registry number, the number of participants, and baseline data. For the studies reported by the same author multiple times, we choose the latest and most complete publication. In addition, we manually searched previous reviews and reference lists of the articles included in our study to find other related studies.  Figure 1 shows a flowchart of the article selection process.

### 2.2. Data Extraction

Each study was revised by two reviewers (CLZ and KF) using Endnote (Vision X9). We used the following inclusion criteria: (1) study on the relationship between EZH2 and the prognosis of NSCLC; (2) publication details available, including disease name, year of publication, and name of the first author; (3) using immunohistochemistry (IHC) or polymerase chain reaction (PCR) to detect the expression of EZH2 in lung cancer tissue; (4) investigating the relationship between EZH2 expression and survival and providing sufficient data to estimate the hazard ratios (HRs) of the survival rate and its 95% confidence interval (CI); (5) the full text is available. If HRs cannot be obtained directly from the original study, the data were calculated using Kaplan-Meier curves according to the method provided by Tierney et al. [11]. Studies were excluded if meeting one of the exclusion criteria: (1) reviews, case reports, conference abstracts, or letter or author corrections to the editor; (2) duplicate articles; (3) animal studies; (4) survival data missing or impossible to calculate. The differences were resolved through discussions with a third investigator (FC). Data collection was conducted using a standardized extraction table.

### 2.3. Quality Assessment

All selected articles were evaluated using the Newcastle-Ottawa scale. This scale includes three aspects: study group selection, study group comparison, and outcome measurement and has a score ranging between 0 and 9.

### 2.4. Bioinformatics Analysis

To further validate and complement this meta-analysis, we analyzed the relationship between EZH2 mRNA expression and NSCLC overall survival (OS) using the Kaplan-Meier plotter database. The Cancer Genome Atlas (TCGA) data of EZH2 transcriptome expression were extracted from TCGA data portal of lung adenocarcinoma and lung squamous cell carcinoma. In addition, we investigated the correlation between EZH2 expression and EGFR gene, KRAS gene, BRAF gene, and smoking in lung cancer patients from TCGA database. The results were considered statistically significant if the *p* value was less than 0.05.

### 2.5. Statistical Analysis

We used Cochran's *Q* test and Higgin's *I*^2^ statistics to evaluate the heterogeneity between pooled studies. If there was no significant heterogeneity (*p* > 0.05 and *I*^2^ < 50%), we would like to use a fixed model; otherwise, we preferred to use a random-effects model. Publication bias was evaluated using funnel plots, Begg's statistical test, and Egger's statistical test. We used the STATA software for statistical analysis (STATA Corporation, College Station, USA, version 12.0).

## 3. Result

### 3.1. Study Characteristics

We retrieved 794 records from the online database using the retrieval strategy provided above. By manually reviewing the titles and abstracts, we rejected duplicates and unavailable articles. Then, we selected 132 articles for full-text screening. Finally, 13 studies were included after cross-reference. These studies were conducted in China, the United States, Denmark, and Japan, and all is aimed at assessing the relationship between EZH2 expression and prognosis in patients with NSCLC. In these studies, EZH2 expression was detected in lung cancer tissues in 2180 cases, including 1064 cases in which EZH2 was highly expressed. The expression of EZH2 was tested by immunohistochemical staining in 12 studies and by a polymerase chain reaction in one study [12]. The HR and 95% CI were obtained directly from the report for six studies [13–18] and obtained from the survival curve for six other studies [12, 19–23]. The remaining study was included, but the HR could not be obtained; therefore, it was analyzed qualitatively only [19]. The characteristics of the included studies are shown in Table 1, and the HR and 95% CI are shown in Table 2.

### 3.2. Qualitative Assessment

The research quality score, as measured with the Newcastle-Ottawa scale, was above 6 for all the studies (Table 3).

### 3.3. Meta-Analysis Results

**Figure 2:**
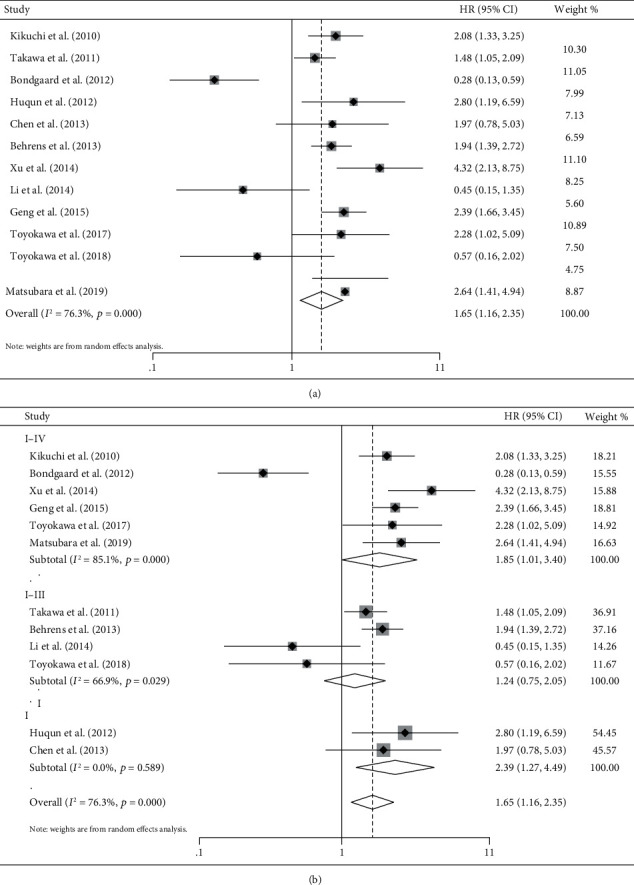
Forest plot of overall survival analysis and disease-free survival analysis. (a) Meta-analysis of EZH2 expression and overall survival. (b) Meta-analysis of EZH2 expression and overall survival in different stages of lung cancer.

Survival analysis was performed with 12 studies we included. Because of the high degree of heterogeneity (*I*^2^ = 76.3% and *p* ≤ 0.001), we chose a random-effects model to compare the low and high expression of EZH2. We found that a high expression of EZH2 indicated poor OS (HR = 1.65and 95% CI 1.16-2.35; *p* ≤ 0.001) (Figure 2(a)). To find sources of heterogeneity between the studies, we performed subgroup analyses. Subgroup analyses showed a high heterogeneity in stages I-IV (*I*^2^ = 85.1% and *p* ≤ 0.001) and in stages I-III (*I*^2^ = 66.9% and *p* = 0.029) (Figure 2(b)). There was no significant heterogeneity in stage I (*I*^2^ = 0.00% and *p* = 0.589). These results suggest that the cancer stage may be a source of heterogeneity. The study that was not included in the meta-analysis [19] also showed that the regulation of EZH2 expression is upregulated in lung cancer and that its expression is positively correlated with cancer stage and lymph node metastasis in patients with lung cancer.

### 3.4. Survival Analysis of Lung Cancer through Kaplan-Meier Plotter Database

**Figure 3:**
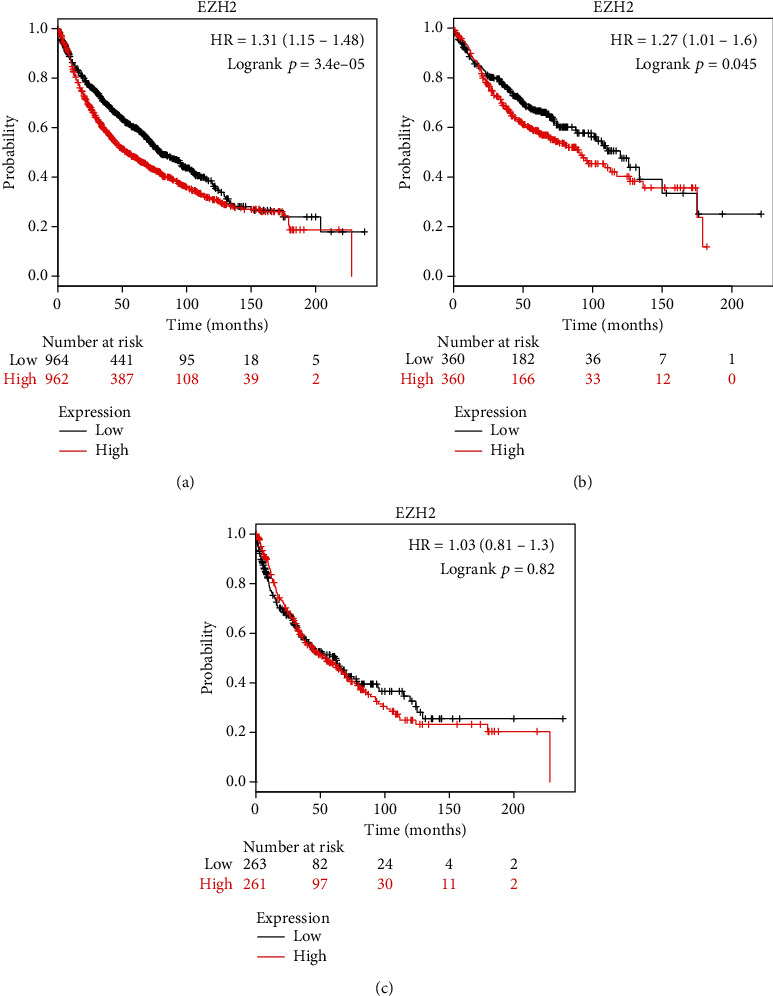
Kaplan-Meier survival curves for lung cancer patients, stratified by EZH2 expression levels: (a) lung cancer, (b) lung adenocarcinoma, and (c) lung squamous cell carcinoma.

In order to verify the results of the meta-analysis, we chose to use the Kaplan-Meier database for survival analysis. Among the NSCLC medical records included in the study, EZH2 mRNA had a high expression in 962 cases with a median survival time of 54.17 months and a low expression in 964 cases with a median survival time of 79.50 months (HR = 1.31and 95% CI 1.15~1.48; *p* < 0.05) (Figure 3(a)). Further analysis showed that 357 cases of lung adenocarcinoma with EZH2 high expression had a median survival time of 90 months and 360 cases with EZH2 with low expression had a median survival time of 119.87 months (HR = 1.27and 95% CI 1.01−1.6; *p* < 0.05) (Figure 3(b)). In 261 cases of lung squamous cell carcinoma, EZH2 mRNA had a high expression with a median survival time of 52.97 months, and in 263 cases, there was a low expression with a median survival time of 62.00 months (HR = 1.03 and 95% CI 0.81−1.3; *p* = 0.82) (Figure 3(c)). Therefore, most of the results from the Kaplan-Meier plotter database analysis are consistent with our meta-analysis; nonetheless, no significant statistical significance was found for lung squamous cell carcinoma. This may suggest that the expression of EZH2 affects the prognosis of patients with lung cancer, and this relation may depend on the pathological type.

### 3.5. EZH2 Expression Analysis through TCGA Database

**Figure 4:**
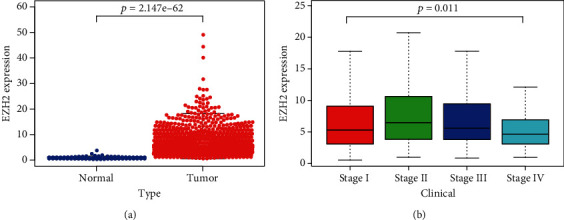
TCGA data analysis. (a) Comparison of EZH2 expression between normal tissue and tumor tissue. (b) Comparison of EZH2 expression between stages of patients with lung cancer.

We selected transcriptome analysis data from patients with lung adenocarcinoma and lung squamous cell carcinoma from TCGA. After data integration, 108 normal specimens and 1037 lung cancer specimens were extracted. The expression of EZH2 and the correlation between the high expression of EZH2 and cancer stage between normal specimens and tumor specimens were compared. The results showed that the expression of EZH2 in lung cancer tissues was significantly different from that in normal tissues (*p* < 0.05) (Figure 4(a)), and there was a significant correlation between EZH2 expression and the cancer stage (*p* < 0.05) (Figure 4(b)).

**Figure 5:**
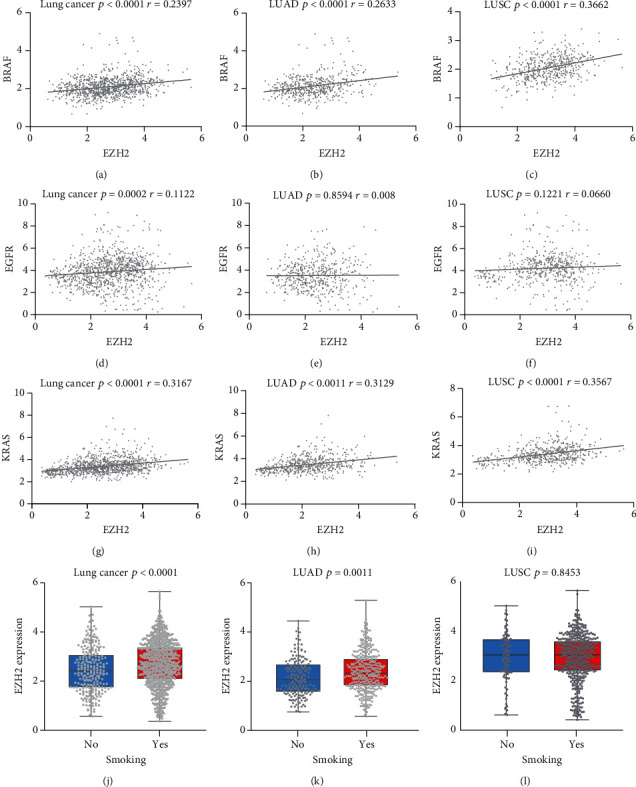
TCGA data analysis. (a) Overall correlation between EZH2 and BRAF in adenocarcinoma and squamous cell carcinoma. (b) Correlation between EZH2 and BRAF in adenocarcinoma. (c) Correlation between EZH2 and BRAF in squamous cell carcinoma. (d) Overall correlation between EZH2 and EGFR in adenocarcinoma and squamous cell carcinoma. (e) Correlation between EZH2 and EGFR in adenocarcinoma. (f) Correlation between EZH2 and EGFR in squamous cell carcinoma. (g) Overall correlation between EZH2 and KRAS in adenocarcinoma and squamous cell carcinoma. (h) Correlation between EZH2 and KRAS in adenocarcinoma. (i) Correlation between EZH2 and KRAS in squamous cell carcinoma. (j) Overall correlation between EZH2 and smoking in adenocarcinoma and squamous cell carcinoma. (k) Correlation between EZH2 and smoking in adenocarcinoma. (l) Correlation between EZH2 and smoking in squamous cell carcinoma. Lung cancer: adenocarcinoma of lung and squamous cell lung carcinoma; LUAD: adenocarcinoma of lung; LUSC: squamous cell lung carcinoma; *r*: coefficient of correlation.

We extracted from TCGA database from lung adenocarcinoma patients (among which 165 were nonsmokers and 361 were smokers) and lung squamous carcinoma patients (among which 86 were nonsmokers and 464 were smokers), 526 lung adenocarcinoma patients and 550 lung squamous carcinoma patients with EGFR expression, 585 lung adenocarcinoma patients and 550 lung squamous carcinoma patients with KRAS expression, and 527 lung adenocarcinoma patients and 502 lung squamous carcinoma patients with BRAF expression. We found that the expression of EZH2 is positively correlated with KRAS (*r* = 0.3167 and *p* < 0.001) and BRAF (*r* = 0.2397and *p* < 0.0001) gene expression in lung squamous cell carcinoma and lung adenocarcinoma (Figures 5(a) and 5(g)). In lung squamous cell carcinoma, EZH2 expression is positively correlated with EGFR expression, but the correlation is weak (*r* = 0.1122 and *p* < 0.001) (Figure 5(f)). TCGA data shows that high expression of EZH2 is related with smoking (*p* < 0.0001) (Figure 5(j)), especially in lung adenocarcinoma (*p* = 0.0011) (Figure 5(k)). The result was not statistically significant in lung squamous cell carcinoma (*p* = 0.8453) (Figure 5(l)).

### 3.6. Sensitivity Analysis

**Figure 6:**
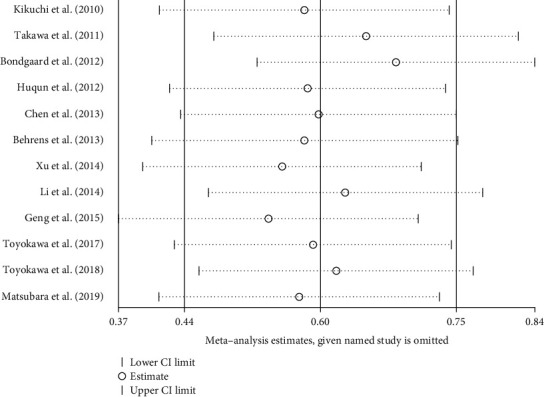
Result of sensitivity analyses by omitting one study in each turn.

To evaluate the stability of the meta-analysis results, we conducted a sensitivity analysis by excluding studies one by one and recalculating the combined HR. We found that no individual study affected the stability of the entire meta-analysis (Figure 6).

### 3.7. Publication Bias Assessment

**Figure 7:**
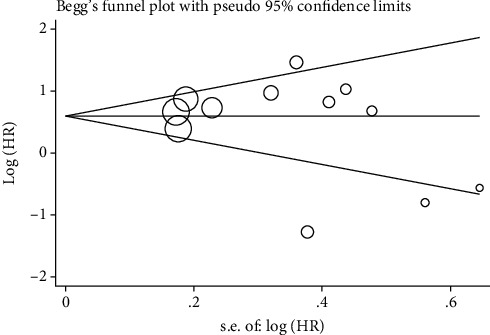
Funnel plot of EZH2 and overall survival.

Begg's funnel plot indicated an absence of risk of publication bias (Table 4 and Figure 7). This was confirmed by Egger's regression intercept (Table 5).

## 4. Discussion

The purpose of this study was to investigate the relationship between the expression of EZH2 and the prognosis of NSCLC and to evaluate the prognostic value of EZH2 in NSCLC. The high expression of EZH2 is closely related to a poor prognosis in many tumors, but its value in the prognosis of NSCLC is still controversial [8, 25, 26]. A total of 13 studies were included in this meta-analysis, with HR as the effect size and the 95% CI upper and lower limits as variables. The meta-analysis showed that high expression of EZH2 in NSCLC tissues indicates a poor prognosis. The analysis of biological information mined by the Kaplan-Meier plotter database and TCGA database is consistent with this result, suggesting that EZH2 may be an independent prognostic factor for NSCLC. To our best knowledge, this is to date the only meta-analysis to estimate the survival rate of patients with NSCLC in which the correlation between EZH2 expression rate and HR is explored. Furthermore, previous meta-analyses did not specifically explain the prognostic relationship between EZH2 and lung cancer [27], or no bioinformatics evidence was used to support it [28]. Therefore, we searched TCGA database and Kaplan-Meier plotter database for large-scale global non-small-cell lung cancer database mining to find EZH2 potential correlation with NSCLC. The Kaplan-Meier plotter database analysis showed that the median survival time of patients with EZH2 mRNA high expression is 54.17 months, and the median survival time of patients with EZH2 mRNA low expression is 79.50 months (HR = 1.31 and *p* < 0.05), suggesting EZH2 high expression in NSCLC tissues indicates a poor prognosis and has significant correlation with cancer stage and pathological type. As single-stranded RNA molecules with a length of 200 to 100,000 nt, long-chain noncoding RNAs have multiple interactions with EZH2. LINC01234 interacts with RNA-binding proteins LSD1 and EZH2, resulting in histone modification and transcriptional suppression of the antiproliferative gene BTG2 [29]. RNA immunoprecipitation and chromatin immunoprecipitation experiments showed that LINC00467 recruited EZH2 to the htrA serine peptidase 3 (HTRA3) promoter, thereby inhibiting the expression of downstream genes of HTRA3 [30]. lncRNA FOXC2-AS1 may inhibit p15 expression by interacting with EZH2, thereby promoting the tumorigenesis of NSCLC [31]. This shows that, through the upregulation of the EZH2 expression, long noncoding RNA silences tumor suppressor genes and promotes lung cancer invasion and migration, which are associated with poor prognosis. This is consistent with our findings. However, the high expression of EZH2 is not only related to the regulation of long noncoding.

RNA, but also to other aspects of epigenetics, such as DNA methylation and histone modification. According to the human ENCODE database (https://www.encodeproject.org/), EZH2 is one of the targets of the polycomb protein and plays an important role in embryo development and tumorigenesis. EZH2 is an important candidate regulatory factor for the aging methyl group and is inhibited in senescent cells, i.e., the share of EZH2 may decrease with age [32, 33]. This indicates that the expression of EZH2 should decrease with age. However, the data we obtained from TCGA indicates that EZH2 is highly expressed in NSCLC even in older adults [29–31, 34, 35]. This shows that the high expression of EZH2 “juvenizes” tumor cells, thereby affecting the prognosis of non-small-cell lung cancer.

The main treatment of patients with NSCLC is chemotherapy, and drug resistance is a major clinical problem. Some studies have suggested that AFAP1-AS1 can activate the PI3K/AKT pathway and interact with EZH2 to inhibit the apoptosis of A549/DDP cells, thereby inducing NSCLC resistance to DDP [36]. The specific interaction of LINC00665 and EZH2 significantly reduces the expression level of phosphorylated AKT (p-AKT) [37]. A study by Zhan et al. [38] showed that cisplatin induces high expression of HOXB13 in lung adenocarcinoma cells. HOXB13 can directly upregulate a series of genes related to metastasis and drug resistance by directly binding to EZH2, promoting tumor progression and predicting poor prognosis. The above studies show that EZH2 is involved in drug resistance in patients with NSCLC, which can lead to a poor prognosis. Surprisingly, EZH2 has also a certain correlation with the body's immunity, i.e., it can promote the development of T cells and B cells [39] and plays an important role in B cell division and activation [40]. In an experiment, it was found that a lentivirus expressing shRNA mediated EZH2 gene knockdown, inhibiting the mRNA and protein expression levels of PD-L1, thereby delaying the progression of lung cancer in vivo by enhancing the antitumor immune response. This indicates that EZH2 overexpression leads to poor prognosis in patients with lung cancer and is positively correlated with the expression of the immunosuppressive molecule PD-L1 [41]. Moreover, Wu et al. [42] experimentally proved that curcumin inhibits the growth and metastasis of lung cancer, partly by inhibiting EZH2. At present, there are many studies on EZH2 inhibitors [43–46], and some drugs have entered clinical research phase. All the above shows that EZH2 is a very promising independent prognostic factor for NSCLC.

Cancer stage and pathological type are important factors influencing the survival of patients with NSCLC [47]. Previous studies have determined the clinical and pathological characteristics of early NSCLC, such as large tumor volume, lymphatic vessel invasion (LVI), or visceral pleural invasion (VPI) characteristics of poor prognosis [48, 49]. Our subgroup analysis also suggests that the cancer stage may be a source of heterogeneity.

The results from the Kaplan-Meier survival curve database also suggest that EZH2 in lung adenocarcinoma predicts a poor prognosis. However, the current staging system is based on anatomical information and is not comprehensive enough in predicting survival outcomes and reflecting NSCLC biological heterogeneity. Therefore, it is very valuable to study noninvasive and easily available pretreatment variables to estimate the survival outcome of lung cancer. It is necessary to further study the prognostic value of EZH2 for NSCLC.

As we all know, smoking is an important risk factors for lung cancer. As the amount of smoking increases, the prognosis of patients also deteriorates [50]. Studies have found that smoking can affect the expression of EZH2 through reduced DAB2IP via H3K27me3 in COPD patients and promote the transformation of COPD into lung cancer [51]. We investigated the relationship between the expression of EZH2 in TCGA database and smoking. The extraction results of TCGA database showed that smoking was related to the high expression of EZH2, especially in lung adenocarcinoma. Moreover, the results of 6 studies included in the review showed that smoking is an exposure factor of EZH2 expression (Table 6). Studies have shown that exposure to tobacco smoke condensate induces increased the expression of EZH2 in cultured lung cancer cell lines [52], and DNA hypermethylation is a common risk factor for smoking behavior in NSCLC patients [53]. Therefore, the high expression of EZH2 may be related to long-term smoking.

Current treatments allow more targeted therapies and immunotherapy into our field of vision. Studies have shown that EZH2 promotes tumorigenesis by methylating the expression of multiple tumor suppressor genes [7]. In non-small-cell lung cancer, we found that the expression of EZH2 is associated with poor prognosis. We further analyzed the expression of EZH2 in EGFR, KRAS, and BRAF-positive patients. The results showed that high expression of EZH2 is often present in KRAS and BRAF-positive patients. Previous research results indicate that RAS is a key downstream effector of epidermal growth factor receptor (EGFR), which is activated by mutation and/or overexpression in a variety of human cancers. B-Raf proto-oncoprotein (BRAF) is a serine/threonine kinase that regulates cell proliferation, differentiation, angiogenesis, and cell death. It plays a role in the downregulation of RAS and sends signals through the MAPK/ERK pathway [54, 55]. We believe that the mechanism of high expression of EZH2 on non-small-cell lung cancer may be related to the KRAS and BRAF pathways. It may be that EZH2 silences the genes that inhibit KRAS and BRAF leading to their high expression, or it may be that KRAS and BRAF promote tumor development by activating EZH2.

According to our results, the correlation between the expression of EZH2 and the expression of EGFR in lung carcinoma is not statistically significant. It means there is no evidence of correlation between the two. Nonetheless, from the perspective of its relationship with KRAS and BRAF, we may think that the mechanism or pathways of EZH2 and EGFR have some similarities, but as far as we know, no relevant studies have confirmed this. Studies have shown that the expression of EZH2 is detected in lung precancerous lesions [56], which indicates that EZH2 may also play a role in the diagnosis of lung cancer. Therefore, we believe that the combined detection of EZH2 with EGFR, KRAS, and BRAF has more advantages in the diagnosis and treatment of lung cancer. EZH2 may be a new target for the treatment of non-small-cell lung cancer. This is only speculative at this stage and further experimental verification is still needed.

EZH2 is not only related to NSCLC, but it is also closely related to the occurrence and development of other tumors. For example, in metastatic prostate cancer, both the content of EZH2 messenger RNA and EZH2 protein are increased. Additionally, studies show that high expression of EZH2 is related to bad prognosis in prostate cancer, and it is also an indicator to distinguish indolent prostate cancer from prostate cancer with lethal evolution [57]. Nikoloski et al. [58] pointed out that EZH2 is the target of various types of deletions and mutations in bone marrow dysplasia (MDS), and EZH2 may play the role of an oncogene. Wang et al. [59] found that lncRNA MALAT1 promotes the development of mantle cell lymphoma by binding to EZH2. In diffuse large B cell lymphoma, McCabe et al. [60] found that inhibition of EZH2 activity may provide a promising treatment for EZH2 mutant lymphoma. However, Schmitz et al. found that diffuse large B cell lymphomas based on EZH2 mutations and BCL2 translocation genetic subtypes have higher survival rates than other subtypes [61]. This also suggests to some extent that EZH2 not only promotes tumor progression but may play a beneficial role in certain tumors or certain tumor subtypes. Depending on the cellular environment and the activated oncogenic pathways, the changes in epigenetic modifications caused by EZH2 defects may lead to tumor progression through various mechanisms.

This is the first meta-analysis investigating the relationship between EZH2 expression and prognosis in patients with NSCLC that is supported by bioinformatics analysis. However, our study suffers from certain limitations. Firstly, we found a significant heterogeneity between the included studies (*I*^2^ = 76.3% and *p* ≤ 0.001). Despite the use of subgroup analysis and sensitivity analysis, the source of heterogeneity cannot be fully traced. Secondly, this study included 13 studies with 2180 patients, of which six had no clear HR values. The part of HRs was estimated from the Kaplan-Meier survival curve, which reduced the credibility of our results. And all the included studies were retrospective designed. Finally, this review is limited to studies published in English. Therefore, publication bias cannot be ruled out.

## 5. Conclusion

Our meta-analysis and bioinformatics analyses show that the high expression of EZH2 indicates poor OS, and EZH2 may be an independent prognostic factor affecting NSCLC. EZH2 high expression or its synergistic action with KRAS and BRAF mutations affects the prognosis of non-small-cell lung cancer. However, these results are based on retrospective studies, which limit the conclusions. Therefore, further research is needed on the effect of EZH2 high expression on the prognosis of patients with NSCLC.

## Figures and Tables

**Table 1:** Main characteristics of the eligible studies.

Study	Country	Method	Antibody Corp	Sample	*N*	*N* of positive cases	Stage	Lung cancer type	Related characteristics
Kikuchi et al. 2010 [16]	Japan	IHC	BD Transduction Laboratories, Mississauga, ON, Canada	Issue	157	95	I–IV	NSCLC (ADC and NADC)	Nonadenocarcinoma histology, moderate and poor differentiation, advanced pathologic tumor classification, and high Ki-67 and cyclin E labeling indices
Takawa et al. 2011 [17]	Japan	IHC	Leica Microsystems, Wetzlar, Germany	Issue	292	135	pt1-pt, pN0-pn2, and pm0	NSCLC	Stage (grade III tumors showed significantly higher EZH2 protein expression than grade I and grade II tumors)
Bondgaard et al. 2012 [20]	Denmark	IHC	BD Transduction Laboratories, San Jose, CA, USA	Issue	96	27	I–IV	LENEC	Grade
Huqun et al. 2012 [15]	China	IHC	Cell Signaling Technology, Danvers, MA	Issue	106	66	I	NSCLC	Tumor size
Chen et al. 2013 [14]	Japan	IHC	Cell Signaling Technology, MA, USA	Issue	42	18	I	NSCLC (22 ADC and 20 SCC)	Male, non-ADC, smoking history, vessel invasion
Xu et al. 2014 [18]	China	IHC	Cell Signaling Inc., Danvers, MA, USA	Issue	360	204	IIIB to IV	NSCLC	Higher TNM, poorer differentiation, nodal metastases
Geng et al. 2015 [21]	China	IHC	Cell signaling	Issue	195	96	I–IV	NSCLC	Tumor differentiation, lymph node metastasis, size and TNM stage
Toyokawa et al. 2017 [22]	Japan	IHC	Leica Biosystems, Newcastle-upon-Tyne, UK	Issue	268	117	I–IV	NSCLC	SUVmax、the presence of vascular invasion and SCC histology
Toyokawa et al. 2018 [23]	Japan	IHC	Leica Biosystems, Newcastle-upon-Tyne, UK	Issue	40	25	I–III	SCLC	None
Matsubara et al. 2019 [24]	Japan	IHC	Takara, Shiga, Japan	Issue	350	182	I–IV	Adenocarcinoma	Male sex, advanced stage, pleural and lymphovascular invasion, and vimentin expression
Behrens et al. 2013 [13]	American	IHC	Mouse monoclonal, NCL-L; Novocastra, Leica Niosystem	Issue			I–III	NSCLC (SCC and ADC)	Age, gender, histology, disease stage, size of tumor, smoking status, EGFR mutation, KRAS mutation
Li et al. 2014 [12]	China	PCR	OriGene Technology	Issue	54	28	I–III	NSCLC	Age, gender, histology, disease stage, size of tumor, smoking status, metastasis, and recurrence
Xu et al. 2013 [19]	China	IHC	Cell Signaling Inc., Danvers, MA, USA	Issue	60	22	I–IV	NSCLC	Lymph node metastasis and TNM stages

IHC: immunohistochemistry; PCR: polymerase chain reaction; NSCLC: non-small-cell lung carcinoma; SCLC: small-cell lung carcinoma; SCC: squamous cell carcinoma; ADC: adenocarcinoma; EGFR: estimated glomerular filtration rate; TNM: tumor node metastasis; *N*: number.

**Table 2:** Summary of HRs and their 95% CI.

Author	Year	HR	LCI	UCI
Kikuchi et al. [16]	2010	2.08	1.32	3.23
Takawa et al. [17]	2011	1.482	1.051	2.091
Bondgaard et al. [20]	2012	0.28	0.13	0.57
Huqun et al. [15]	2012	2.8	1.19	6.59
Chen et al. [14]	2013	1.975	0.775	5.031
Xu et al. [19]	2014	4.32	2.13	8.73
Geng et al. [21]	2015	2.39	1.66	3.46
Toyokawa et al. [22]	2017	2.28	1.02	5.09
Toyokawa et al. [23]	2018	0.57	0.16	2.01
Matsubara et al. [24]	2019	2.64	1.41	4.94
Behrens et al. [13]	2013	1.943	1.387	2.723
Li et al. [12]	2014	0.45	0.15	1.35

CI: confidence interval; HR: hazard ratio; LCI: low confidence interval; UCI: upper confidence interval.

**Table 3:** Quality assessment based on the Newcastle-Ottawa scale.

Study	Year	Selection	Comparability	Outcome	Total score
Kikuchi et al. [16]	2010	4	2	2	8
Takawa et al. [17]	2011	3	2	2	7
Bondgaard et al. [20]	2012	4	1	2	7
Huqun et al. [15]	2012	3	2	2	7
Chen et al. [14]	2013	3	1	3	7
Xu et al. [19]	2013	4	2	3	9
Behrens et al. [13]	2013	4	2	2	8
Xu et al. [18]	2014	4	1	2	7
Li et al. [12]	2014	3	1	2	6
Geng et al. [21]	2015	3	2	2	7
Toyokawa et al. [22]	2017	3	2	2	7
Toyokawa et al. [23]	2018	3	2	2	7
Matsubara et al. [24]	2019	3	2	2	7

**Table 4:** Begg's test.

Begg's test
adj.Kendall′s score (*P* − *Q*) = −16
Std.Dev.of score = 14.58
Number of studies = 12
*z* = −1.1
Pr > ∣*z* | = 0.273
*z* = 1.03 (continuity corrected)
Pr > ∣*z* | = 0.304 (continuity corrected)

adj.: adjusted; Std. Dev.: standard deviation.

**Table 5:** Egger's test.

Egger's test						
Std_Eff	Coef.	Std. err.	*t*	*p* > ∣*t*∣	(95% Conf. interval)	
Slope	0.930	0.401	2.320	0.043	0.036	1.824
Bias	-1.331	1.464	-0.910	0.385	-4.592	1.93

**Table 6:** Correlation between EZH2 and smoking in six studies.

Study	OR	EZH2 high		EZH2 low	
		Yes	No	Yes	No
Kikuchi et al. 2010 [16]	8.69	74	11	24	31
Takawa et al. 2011 [17]	3.64	114	21	94	63
Chen et al. 2013 [14]	4.90	14	4	10	14
Geng et al. 2015 [21]	1.02	50	46	51	48
Toyokawa et al. 2018 [23]	5.50	22	1	12	3
Li et al. 2014 [12]	0.23	10	12	25	7
